# Targeting the gap of planetary health education in medical teaching: A student-led initiative develops the course “Klima-LIMETTE” on climate change and health using simulated patients

**DOI:** 10.3205/zma001772

**Published:** 2025-09-15

**Authors:** Kyra Lilier, Kate Bärnighausen, Thorsten Kuczius, Veronika K. Jaeger, Alicia Basoglu, André Karch, Tom Theiler, Alberta Ajani, Eva-Maria Schwienhorst-Stich, Helmut Ahrens

**Affiliations:** 1 Heidelberg University, Faculty of Medicine and University Hospital, Heidelberg Institute of Global Health (HIGH), Heidelberg, Germany; 2 University of Münster, Institute of Hygiene, Münster, Germany; 3 University of Münster, Institute of Epidemiology and Social Medicine, Münster, Germany; 4 University of Münster, Institute for Medical Microbiology, Münster, Germany; 5 University of Münster, Institute for General Medicine, Münster, Germany; 6 University Hospital Würzburg, Department of General Practice and Family Medicine, Würzburg, Germany; 7 University of Münster, Medical Faculty, Institute of Education and Student Affairs, Münster, Germany

**Keywords:** planetary health education, medical education, climate change and health, best-practice example

## Abstract

**Background:**

Planetary health education highlights the growing impact of climate change on human health – an urgent and relevant issue for healthcare providers that remains inadequately addressed in medical education.

**Method:**

A student-led initiative at the University of Münster, Germany, has developed the “Klima-LIMETTE” (Engl.: “Climate-LIMETTE”), a course that teaches the health implications of climate change. It builds on the established infrastructure “LIMETTE” (Lernzentrum für individualisiertes medizinisches Tätigkeitstraining, Engl.: Learning center for individual medical skills training), that uses medical scenarios with simulated patients. Scenarios were developed based on current research on the effects of climate change on health with a focus in Germany. An additional blended e-learning course was designed to convey the knowledge needed for the case simulations and to promote a comprehensive understanding of planetary health.

**Results:**

The “Klima-LIMETTE” was conducted twice as a pilot study with 32 students. The cases were evaluated to be realistic and relevant. Students ranked the “Klima-LIMETTE” as “good” or “very good” on a six-point Likert scale.

**Conclusion:**

Health-relevant climate information can be presented practically and theoretically in medical education. This course acts as a best-practice example of Planetary Health Education in medical teaching through interdisciplinary cooperation. The course is now implemented in the curriculum and jointly organized by four complementary institutes within the University of Münster.

## Zusammenfassung

**Hintergrund:**

Die planetare Gesundheitslehre thematisiert die zunehmenden Auswirkungen des Klimawandels auf die menschliche Gesundheit – ein dringendes und relevantes Thema für Gesundheitsexpert:innen, das in der medizinischen Ausbildung noch nicht ausreichend behandelt wird.

**Methode:**

Eine von Studierenden geleitete Initiative an der Universität Münster hat die „Klima-LIMETTE“ entwickelt; einen Kurs, der die gesundheitlichen Auswirkungen des Klimawandels vermittelt. Sie baut auf der etablierten Infrastruktur „LIMETTE“ (Lernzentrum für individualisiertes medizinisches Tätigkeitstraining) auf, die medizinische Szenarien mit Schauspielpatient:innen darstellt. Die Szenarien wurden auf der Grundlage aktueller Forschung zu den Auswirkungen des Klimawandels auf die Gesundheit mit Schwerpunkt in Deutschland entwickelt. Ein zusätzlicher Blended-E-Learning-Kurs wurde konzipiert, um das für die Fallsimulationen benötigte Wissen zu vermitteln und ein umfassendes Verständnis der planetaren Gesundheit zu fördern.

**Ergebnisse:**

Die „Klima-LIMETTE“ wurde zweimal als Pilotstudie mit 32 Studierenden und zweimal curricular im neunten Semester durchgeführt. Die Fälle wurden als realistisch und relevant bewertet. Die Studierenden bewerteten die „Klima-LIMETTE“ auf einer sechsstufigen Likert-Skala mit „gut“ oder „sehr gut“.

**Schlussfolgerung:**

Gesundheitsrelevante Klimawandelfolgen können in der medizinischen Ausbildung praktisch und theoretisch vermittelt werden. Dieser Kurs dient als Best-Practice-Beispiel dafür, wie planetare Gesundheitslehre in der medizinischen Lehre durch interdisziplinäre Zusammenarbeit verankert werden kann. Der Kurs ist im Curriculum implementiert und wird von vier sich ergänzenden Instituten der Universität Münster gemeinsam organisiert.

## 1. Introduction

The effects of climate change on health such as heat-associated illnesses, air quality-associated diseases, emerging infectious diseases, psychological reactions, and nutritional consequences have become increasingly relevant in everyday medical practice [1]. Prevention measures, the preparedness of healthcare facilities and targeted strategies for risk groups play a critical role for future population health [2]. In Germany, the population is especially vulnerable to heat waves and flooding [2]. In 2022 alone, over 8,000 heat-related deaths were recorded [3]. Healthcare facilities struggle to protect vulnerable groups [4]. The 2021 flood in the Ahr valley and the 2024 flood in Bavaria had impacts on the physical and mental health of the affected population and disrupted healthcare services [5].

Climate change also contributes to increased incidences of allergies to pollen, putting additional stress on the population and the healthcare system [6].

Planetary Health considers the interrelationships between health and the political, economic, social and natural systems. This includes the treatment and prevention of climate-sensitive diseases, as well as the interconnectedness of health with biodiversity loss, pollution, and potential co-benefits for 21^st^-century medicine [7]. Among these co-benefits are plant-based diets and active travel (such as walking and cycling), which support both human health and the planet’s resources [7]. Knowledge of the treatment and prevention of climate-associated diseases is important for adaptation to climate change and preventive action [8]. Prevention of heat-associated illnesses can, for example, have a broad impact that not only protects those affected, but also reduces the overall burden on the healthcare system [9]. These diverse health impacts of climate change influence the daily work of health professionals. However, teaching facilities for medical professionals are slow in adjusting and extending their curricula [8], [10]. In order to cope with these tasks and responsibilities, there are strong calls to prepare students in their training and incorporate planetary health education (PHE) in medical curricula [8], [11], [12]. PHE will “equip and enable learners to drive transdisciplinary and mutually reinforcing actions to protect and restore planetary health” [13]. Aspects of PHE are incorporated in the National Competence-Based Learning Objectives Catalog Medicine 2.0 (NKLM) of Germany, which will become the nationwide basis of the mandatory core curriculum in medical studies [14].

### 1.1. Status quo of medical teaching in PHE in Germany

While some level of teaching exists at many universities in Germany, it is often confined to a few hours in the curriculum and is mainly restricted to elective courses, and is not present in the core curriculum [15]. Further studies on implementation of and initiatives on PHE are currently underway [16]. The “planetary health report card (PHRC)”, a metric-based tool to evaluate PHE in health professional education, and implemented at many medical schools worldwide [17], describes an average result of “D” (A-F) in 2022 [18] among eight German universities [18].

## 2. Project description

### 2.1. Background

Since 2019, students from the local “Health for Future” (H4F) group in Münster, Germany have made efforts to integrate PHE into medical teaching, e.g. through the clinical elective called diagnosis climate crisis. As this was an elective course, it mainly attracted students with prior knowledge or a strong interest in health and climate change. As a preparation for assessing the needs and gaps in PHE at the University of Münster, students from the H4F group (lead by the first author KL) conducted the planetary health report card survey in the academic year 2021/2022. This validated, metric-based evaluation tool asses areas of curriculum, research, community outreach and advocacy, support for student-led initiatives, and sustainability [17]. For the evaluation of the curriculum, the students reviewed teaching materials such as lecture slides, surveyed lecturers and members of the teaching department (IfAS) including the dean of studies via email, reviewed guidelines and asked students from different semesters about their experiences. Within the scope of this interdisciplinary survey, it was found that PHE teaching at the Medical Faculty of Münster is insufficient, overall graded with a “D” (20-39%) in a grading system from A to F [19]. Results show that there are a few planetary health topics in the curriculum, but that the majority of topics are not yet included in the compulsory curriculum. Based on these findings and in order to create a mandatory course for the curriculum, the student initiative, led by two medical students (including the first author KL) developed the practical course “Klima-LIMETTE” (Engl.: “Climate-LIMETTE”), that addresses climate impacts on health using relevant cases for everyday medical practice. They were supported by Dr. med. Helmut Ahrens, the medical director of the learning center “LIMETTE” and the Institutes of Hygiene, General Medicine, Epidemiology and Social Medicine, and Medical Microbiology. 

### 2.2. The teaching tool “LIMETTE” at the Medical Faculty of Münster 

As a format for the new course, the students chose the “LIMETTE” (Engl.: “Lime”) (**L**ernzentrum für **i**ndividualisiertes **m**edizinisches **T**ätigkeits**t**raining, Engl.: Learning center for individual medical skills training), an infrastructure of the Medical Faculty of Münster, that is used for formative assessment in medical education by more than 10 other departments such as the Departments of General Medicine, Neurology and Emergency Medicine. 

The “LIMETTE” provides an OSCE (Objective Structured Clinical Evaluation) -based training on a formative assessment basis [20], [21]. Students perform tasks in different roles (e.g. as a student, resident, neighbor) in a sequential scenario with simulated patients [22], [23]. Patients act as partners to trigger interpersonal skills which help to assess a supervision level of Entrustable Professional Activities (EPAs) (see table 1 (Tab. 1)) based on the EPA Toolkit by the Association of American Medical Colleges [24]. An EPA is defined as a circumscribed activity of professional practice that is entrusted to a learner for independent and unsupervised performance, only if the learner has the required competencies [24].

Feedback is based on the EPA abridged Toolkit [24]. There are checkboxes for the assessment of students progression towards successful integration of their clinical skills [25], [26]. Objective, observable behavior is measured in the myepas.uni-muenster.de database. The Global Rating Freetext function allows for a SWOT analysis to identify specific measurable objective behaviors which are strengths or weaknesses and can be referred to the Core EPA Guide. Opportunities are provided to encourage behavior correction. The supervision level is assessed using the Chen scale (see table 2 (Tab. 2)) [27].

**Figure 1:**
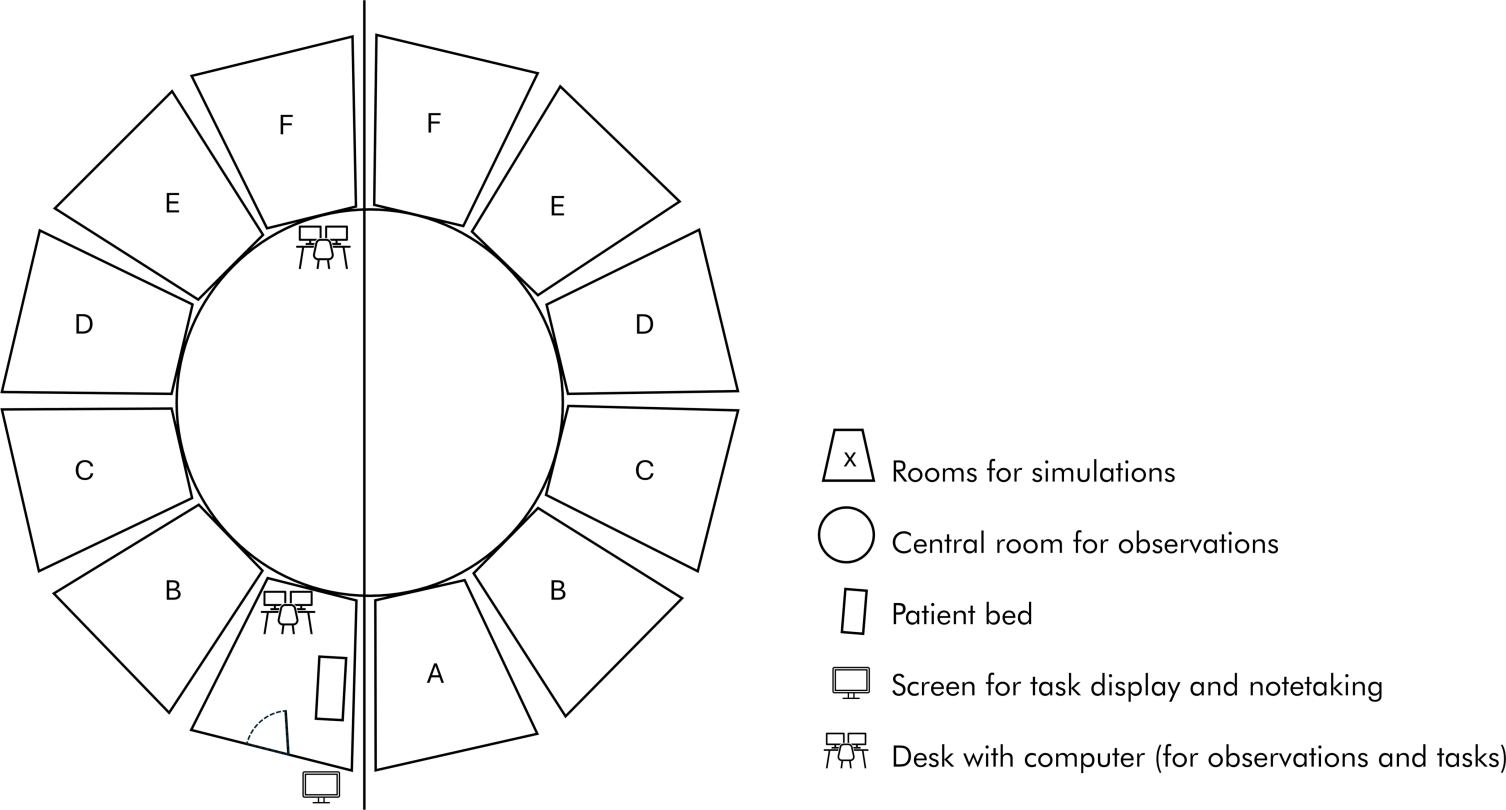
Schematic drawing of the “LIMETTE” building Whilst observers can follow the simulations in all rooms from the larger central room, students from within the room cannot see the observers through semi-transparent mirrors. Observers are equipped with a computer to record the feedback and with earphones to listen to the conversation inside. The simulation rooms have a patient bed for physical examination and a computer, allowing the students to take notes. Next to the front door on the outside of the simulation room a screen displays the task and can be used for adding notes. There are two floors within the building with 12 rooms each to hold the six case scenarios A-F. Students rotate through all six simulations, coordinated by speakers according to the designated time. Camera recordings provide material for debriefing if requested in individual training. The students have 12 minutes to work through and document the different clinical case scenarios, which have to be solved either on the basis of provided documents (paper-based) or through interaction with simulated patients.

In addition, students complete an EPA-based self-assessment before and after working through the cases to encourage reflection on their own abilities. The “LIMETTE” takes place in a specially designed building shaped like a lime when viewed from above (see figure 1 (Fig. 1)).

The training of the observers of the “LIMETTE” is offered within the framework of the medical didactic training and counselling concept “mediCo” and is based on the criteria of the North Rhine-Westphalian State Academy for Medical Education.

For each scenario, a detailed profile of a fictitious patient is created, including their medication intake, history and symptoms using standardized instruments [28]. The simulated patients receive extensive, standardized training about their role, the scenario and the expectations of their behavior during the course [23], [29]. Rehearsals before the course ensure refinement and standardization [29].

### 2.3. Development of the “Klima-LIMETTE” 

**Figure 2:**
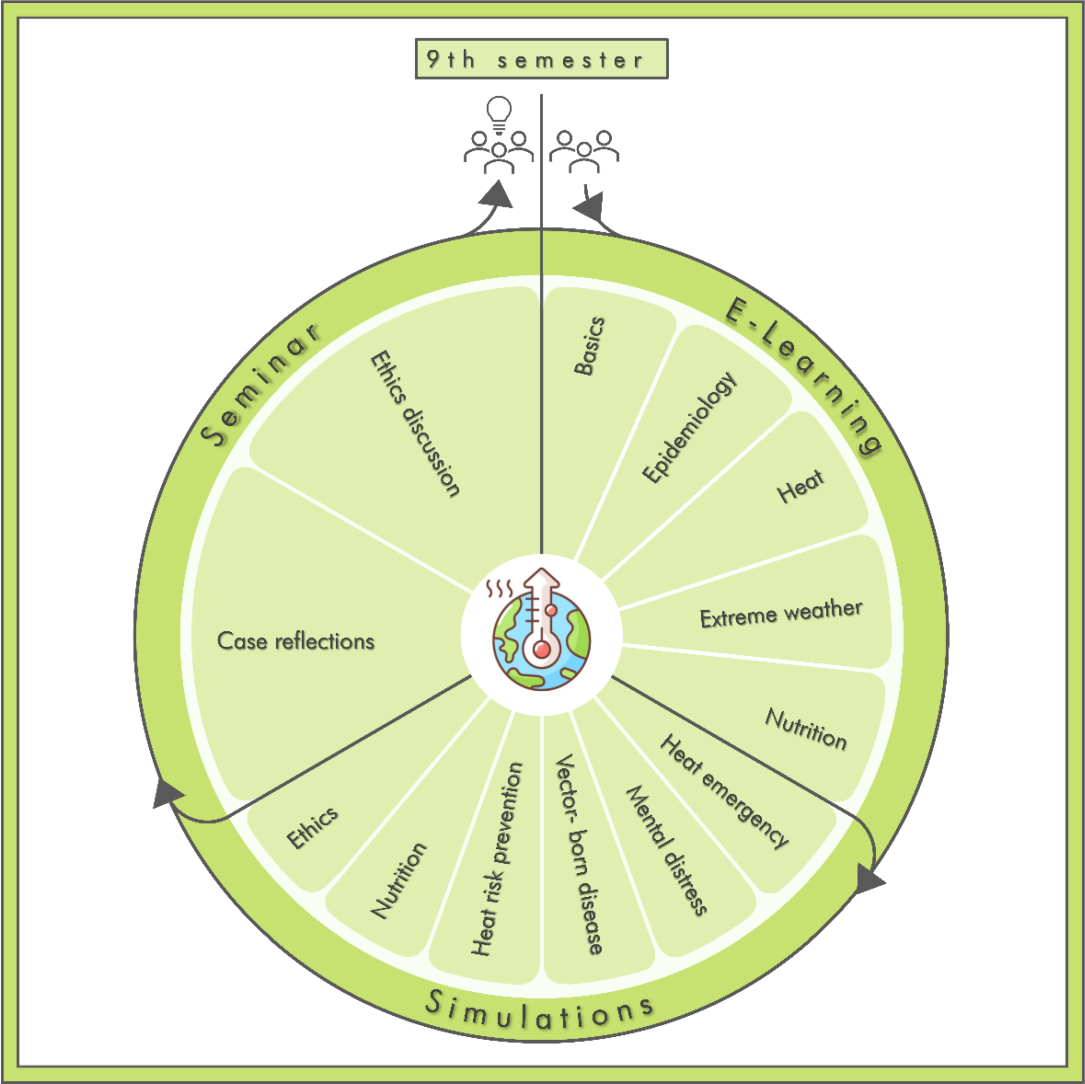
Overview over the components and content of the “Klima-LIMETTE” The “Klima-LIMETTE” (Engl.: “Climate-Lime”) (Lernzentrum für individualisiertes medizinisches Tätigkeitstraining, Engl.: Learning center for individual medical skills training), comprises three components, which can be completed in approximately 90 minutes respectively. Students from the 9^th^ semester will first complete the e-learning to gain skills and knowledge necessary to work through the case simulations with simulated patients. Afterwards, they discuss the cases and ethical implications in a seminar. The aim of the course is to equip students with the knowledge and skills of PHE most relevant to Germany. Here, the topics of each component are displayed in an abstracted lime, referring to the established learning format “LIMETTE” which the course uses to introduce PHE to the Medical Faculty of the University of Münster. The circular makeup of the figure highlights that the “Klima-LIMETTE” is created as a comprehensive, cohesive course where students familiarize themselves with Planetary Health (represented by the symbol in the center), act on their knowledge and discuss implications.

The entire course “Klima-LIMETTE” makes use of the established learning format “LIMETTE”, filling it with PHE. It is composed of three components: a flipped classroom course [30] with an e-learning module within the ILIAS^®^ online platform [31], which teaches the theoretical background for the practical case simulations as blended learning, the simulations in the “LIMETTE” and a subsequent seminar for debriefing and discussion [32] (see figure 2 (Fig. 2)).

**Figure 3:**
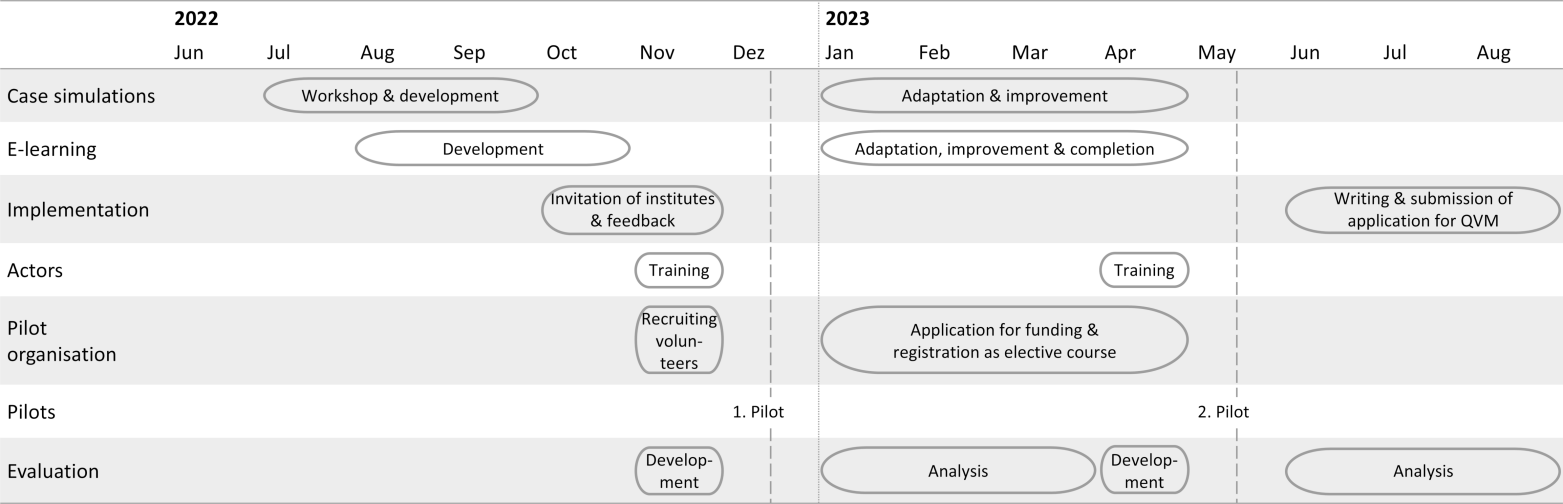
Phases of development and implementation of the “Klima-LIMETTE” This figure details the phases of development and implementation of the course displayed for different components of course development. Case simulations and e-learning materials were developed before and refined after the pilots. Implementation refers to all activities that led to long-term, curricular execution. Actors, pilot organization and pilots display activities for testing the course. The evaluation component was developed before the pilots and analyzed afterwards.

After developing the course, the student initiative invited relevant institutions within the Faculty of Medicine to join the project (see figure 3 (Fig. 3)). The Institutes of Hygiene, General Medicine, Epidemiology and Social Medicine, and Medical Microbiology agreed to build partnerships for the thematically fitting cases and the corresponding areas in the e-learning course, checked the content and methodological quality and made suggestions for improvement.

#### 2.3.1. E-learning course

To close the gaps of PHE in the curriculum and provide the required knowledge for the simulations of the “Klima-LIMETTE”, we designed an e-learning course on the online platform ILIAS^®^. The module aims to promote a comprehensive understanding of the relationship between climate change and health. It is designed to be interactive via the use of graphics and videos and includes questions for self-examination and reflection. Thematically, it is divided into 5 chapters (see table 3 (Tab. 3)). Topics of this course were chosen based on the IPCC Report 2022 [1], the cases in the “Klima-LIMETTE”, and for their relevance for health services in Germany. Students should take approximately 90 minutes to complete the e-learning course. 

#### 2.3.2. Cases

We developed scenarios that depict clinically relevant and realistic situations in a workshop using the CriticalIincident Technique [33], [34]. We chose planetary health topics that were translated into case scenarios based on the IPCC Report 2022 [1], and their relevance for healthcare services in Germany. Learning objectives were defined and determined for each simulation setting. The cases were mapped to an EPA for assessment. Additionally, we reviewed the “National Competence Based Catalog of Learning Objectives for Undergraduate Medical Education (NKLM)” [14], to back up the cases thematically. The cases were developed using a standardized instrument to describe the scenarios in detail, including description of the scene, first sentence and a possible end of scene to allow detailed translation into action [28]. Cases target the topics of heat, psychological consequences of extreme weather events, sustainable nutrition, allergies, vector-borne diseases, and ethical considerations (see attachment 1 ). This ethics case is paper-based, meaning students have to solve the case by reviewing documents. This case is meant to encourage reflection and is not assessed. We developed seven cases, out of which six were chosen for the course. Case six (allergies) was evaluated to be less relevant to be presented in a simulation since it is sufficiently covered in the e-learning module. 

#### 2.3.3. Seminar

After the simulation cases, a 90-minute seminar is held to clarify questions. Lecturers from the participating institutions are on site to briefly go over the most important aspects of the cases. The seminar serves as an opportunity for students to exchange information about the cases they have worked on and compare their skills and experienced challenges with fellow students. Apart from this standardized reflection module, the seminar of the “Klima-LIMETTE” aims to facilitate a discussion about the socio-political role of doctors in relation to preserving the foundation for life. The basis builds the “ethics case”, in which the students are asked to decide whether and how they support a citizen initiative. Students are encouraged to discuss to what extent the preservation of livelihoods, socio-political statements and informing their own patients about health consequences of climate change as well as health co-benefits of a more sustainable lifestyle can or should be part of their daily work. The students are empowered to use their expertise on health in socio-political discussions, strengthening their confidence in contributing with validated knowledge in their field of expertise. 

## 3. Results

### 3.1. Pilots

The first pilot of the “Klima-LIMETTE” was conducted on December 13^th^ 2022 with 8 voluntary students. The second pilot was conducted on May 25^th^ 2023 with 24 students, which is the full number of participants possible in a “LIMETTE” course. This pilot was held as part of an elective course. Participating students ranged from 6^th^ to 9^th^ semester.

### 3.2. Evaluation

The first and second pilot were evaluated by participants using evaluation sheets with free text options (see attachment 2 and attachment 3 ). The short evaluation of the first pilot included a questionnaire filled in on site and mainly referred to the students’ assessment and suggestions for the cases and e-learning improvement. The first pilot was rated as “good” or “very good” (n=7) and students reported the clinical relevance of the selected cases and noted their increased interest in the topic. Free text evaluation revealed suggestions for improvement of the e-learning course, such as more detailed content as well as more content-based discussion in the seminar (see attachment 2 ). The evaluation of the second pilot included a pre- and post-course evaluation form with identical questions. Additionally, the post evaluation included questions about the change of motivation and interest, the preparatory material, the simulations and the seminar, as well as free text commentary options. The average grading was 1,7 (1=very good, 6=unsatisfactory, n=21). Pre- and post- course evaluation showed increased consent to statements such as “the development of the climate crisis affects our clinical routine and our patients” or “as a doctor, I see it as my duty to inform my patients about the health consequences of climate change”. The post-evaluation revealed increased interest in PHE and higher awareness for clinical relevance. The e-learning component was reported to be comprehensible and adequate. The cases were reported to be realistic and useful in preparation for future clinical work, though some cases were mentioned to be overwhelming. This, however, is common feedback from students after LIMETTE-course in other disciplines and may be related to the format rather than the content of the simulations. Additionally, students in both pilots were from higher and lower semesters, with the latter being less trained in some clinical disciplines, as explained by a student in the free text evaluation. Students reported that the seminar deepened their insights, and some appreciated a non purely content-based discussion of the cases with critical reflection of socio-political roles. In the free text evaluation (n=7) students expressed their fondness for the course and some suggested more details in the preparation (see attachment 3 ). We emphasize that the students participated voluntarily and therefore, evaluation is biased.

Both pilots were evaluated verbally by lecturers, simulated patients and trainers. The feedback was documented and shared with all involved. The first implemented, curricular “Klima-LIMETTE” was evaluated using the evaluation tool EVALuna [35]. The evaluation results are pending and will allow a representative evaluation after a two-year test phase.

### 3.3. Implementation

To implement the “Klima-LIMETTE” in the mandatory curriculum at the University of Münster and ensure funding, we applied for QVM (quality improvement funds in teaching) to the Dean’s Office of the Medical Faculty of Münster, and were accepted in December 2023. The Institutes of Hygiene, General Medicine, Epidemiology and Social Medicine, and Medical Microbiology have agreed to cooperatively teach the “Klima-LIMETTE”, which involves creation of teaching capacities and providing human resources for observation and the seminar. The first curricular course was conducted in November 2024 with all students in the 9^th^ semester.

## 4. Discussion

To the best of our knowledge, teaching the health impacts of climate change in this format is unique in Germany to date. Due to the practical and innovative nature of the course, the clinically relevant selection of case studies and the interaction with simulated patients, students can directly put their acquired knowledge into practice.

Regarding the range of PHE, we note that while this course – specifically the e-learning component – aims to provide an overview on planetary health, its focus is on topics that are most relevant for Germany. We acknowledge that the course does not teach all the components of PHE, as multiple frameworks exist. Compared to the PHRC core curriculum topics [17] and other frameworks of education [10], the course falls short on the integration of indigenous knowledge, biodiversity and ecosystems, food and water security and the role of the health system in contributing to climate change. Communicating planetary health is not directly taught or mentioned as a learning objective in the e-learning course (e.g. by introducing the climate sensitive consultation) but is enacted within the simulations and the seminar. Wabnitz et al. propose an even wider approach to PHE in their “National Planetary Health learning objectives for Germany”, including policy, movement building, systems thinking, governance and history, to name a few [12]. In order to create a feasible, acceptable and an easy to incorporate course, we included the most important health consequences of climate change with the focus on Germany, which are not taught in other courses at the University of Münster. Once implemented, the course might pave the way for more controversial and overarching sociological topics to be included in the curriculum, such as human-nature relationships, its history, societal perspectives on planetary health, biodiversity and ecosystems or policy.

Ethical and political dimensions are deeply embedded in PHE, as laid out in the AMEE consensus statement on planetary health and education for sustainable health care [8]. Other authors also identified ethical dimensions as part of PHE [10] and there is a call for educating health professionals in eco-ethical leadership [36]. The “National Planetary Health learning objectives for Germany” state that “graduates reflect on their responsibility to maintain and foster health and the natural and societal systems on which it depends” [12] and even “graduates describe and demonstrate skills to stimulate and implement transformative change in healthcare and other sectors of society” [12] as part of leadership skills. The professional code of conduct for physicians working in Germany that defines the “preservation of the natural foundations of life with regard to their importance for human health” as a medical task additionally offers a national perspective and entry point for discussion [37]. The “Klima-LIMETTE” incorporates these objectives in the ethics case. It is intended to serve as a starting point of reflection where medical professionals should engage in socio-political action. Since there is no definitive right or wrong answer to solving the case, we plan to conclude the seminar with an open discussion of the arguments, allowing each individual to form their own decision. Staff experiences in teaching and discussing professional ethics and research indicates that ethics can be a controversial topic to teach to medical students. This may be associated with disinterest or reluctance to engage [38]. Other research shows that students can be drivers to integrate climate change ethics in their curriculum [9]. Given the political controversial debate in Germany in targeting climate change, political neutrality must be maintained, and the discussion should be held on a professional, health-focused level. Otherwise, acceptance for the respective case and the seminar discussion might not be given. The seminar is meant to sensitize students for their field of expertise and empower them to engage from it and use their knowledge on climate change and health.

A long-term evaluation of the course will determine whether students consistently rate it as valuable and useful or if participation bias, due to its elective nature, influences these ratings. We will continue to improve the course based on its evaluation to ensure high quality PHE.

### 4.1. Adaptability & uptake

As few medical teaching facilities in Germany have an infrastructure like the “LIMETTE” with simulated patients, other ways to teach the content of this course will need to be established. Cases could be discussed and taught in seminar form, with a paper-based version including pictures or videos. Practical skills assessed in the “Klima-LIMETTE”, such as physical examination, are not PHE-specific, and could be optionally included. Alternatively, case vignettes could be taught in an e-learning format with videos of scenarios or virtual patients. If capacity to build new courses is limited, the cases and respective background knowledge from the e-learning could be incorporated into the existing courses and lectures of the respective institutions. We feel that the interdisciplinary approach of the “Klima-LIMETTE” which involves multiple institutions, has enhanced the quality of teaching content while also fostering collaboration within each institution’s scope and capacities. Teaching PHE could also build on existing courses, such as the Planetary Health Academy [39], a lecture series organized by “Deutsche Allianz Klimawandel und Gesundheit e.V.” or e-learning courses, such as the course “Planetary Health” by the Virtual University Bavaria [40].

To promote the uptake of PHE at teaching facilities, the Planetary Health Report Card [17] can be a starting point, as it shows gaps in the curriculum, but also existing PHE to build on. The National Competence-Based Learning Objectives Catalog Medicine 2.0 (NKLM) of Germany [14] includes many PHE topics, so incorporating them will be necessary in the future. Another hurdle to uptake of the PHE might be that lecturers are not adequately trained to deliver PHE. However, the Planetary Health Alliance has collected a wide range of materials for PHE and facilitates a PHE community [41], and frameworks and guidelines have been developed to facilitate PHE at teaching facilities [8], [12], [13], [42], [43], [44], [45], [46]. Consistent with recent studies, we have observed strong interest in the course and materials from both teaching staff and students across Germany, highlighting a growing motivation for the uptake of PHE [11], [47]. In line with the principle of resource sharing, we hope this description encourages similar initiatives at other institutions and fosters further collaboration in PHE to better address the needs and interests of medical professionals.

Finally, the “Klima-LIMETTE” is an example of how PHE can be taught at universities as a course, but ultimately, a longitudinal implementation of PHE is the goal with disciplines across semesters engaging with the wide range of PHE topics to enhance learning success [10].

## 5. Conclusion

A student-led initiative at the University of Münster developed and implemented a course on planetary health. The course uses cases with simulated patients to practically convey knowledge and skills, as well as a blended e-learning tool and a seminar to discuss the simulation cases afterwards. To our knowledge, teaching planetary health in such a course format is unique in Germany. The course shows that through interdisciplinary cooperation, planetary health topics can be presented practically as well as theoretically in medical training. This course can act as a best-practice example and guide for other teaching facilities to incorporate planetary health education. By incorporating the “Klima-LIMETTE” into the mandatory curriculum of the Münster medical faculty, we aim to ensure that the climate and health crisis receives the attention it deserves.

## Notes

### Funding

This project was funded by the MedAlum Münster e.V. (pilots) and through ‘Qualitätsverbesserungsmittel’ (on April 30, 2011, the Act to Improve the Quality of Teaching and Studies at North Rhine-Westphalian Universities (Studiumsqualitätsgesetz) came into force. The law includes a guarantee of funds for at least 249 million euros per year, which will flow from the state to the universities in North Rhine-Westphalia as compensation for the abolition of tuition fees from the winter semester 2011/2012. These quality improvement funds, so-called tuition fee replacement funds, will be distributed to the universities according to the number of students in one and a half times the standard period of study and must be used for the specific purpose of improving the quality of studies and teaching. They are allocated to the universities in addition to their basic funding. It is ensured that the funds do not lead to an increase in admission capacity, but are used for additional staff, such as teaching staff and tutors 

[https://recht.nrw.de/lmi/owa/br_text_anzeigen?v_id=10000000000000000250].

### Authors’ contributions

All authors have contributed to the development of the course and manuscript.

### Authors’ ORCIDs

- Kyra Lilier: [0000-0003-4990-9955]
- Kate Bärnighausen: [0000-0002-4466-8921]
- Thorsten Kuczius: [0000-0002-1373-8763]
- Veronika K. Jaeger: [0000-0002-6913-0976]
- Alicia Basoglu: [0000-0001-8993-1159]
- André Karch: [0000-0003-3014-8543]
- Tom Theiler: [0009-0006-7540-0991]
- Alberta Ajani: [0000-0001-5917-9904]
- Eva-M. Schwienhorst-Stich: [0000-0002-7715-5022]
- Helmut Ahrens: [0009-0003-1730-9629]

### Ethics approval

Ethical approval was not needed for this project as the survey was conducted anonymously (Ethikkommission Westfalen Lippe, reference 2023-353-f-N).

## Acknowledgements

The authors would like to thank the MedAlum Münster e.V. for their financial support, the voluntary participants for taking part in the course and their valued feedback and all students from Health For Future Münster who, through their voluntary engagement, developed this course. The student initiative acknowledges the support of the Faculty of Medicine at Münster University, especially the Institute of Education and Student Affairs and the Institutes of Hygiene, General Medicine, Epidemiology and Social Medicine, and Medical Microbiology. For the publication fee we acknowledge financial support by Heidelberg University through the open access publication fund.

## Competing interests

The authors declare that they have no competing interests. 

## Supplementary Material

Case scenarios

Analysis of the evaluation of the 1st pilot, winter semester 2022, n=8

Analysis of the evaluation of the 2nd pilot, 1st trail as elective course, summer semester 2023, n=24

## Figures and Tables

**Table 1:**
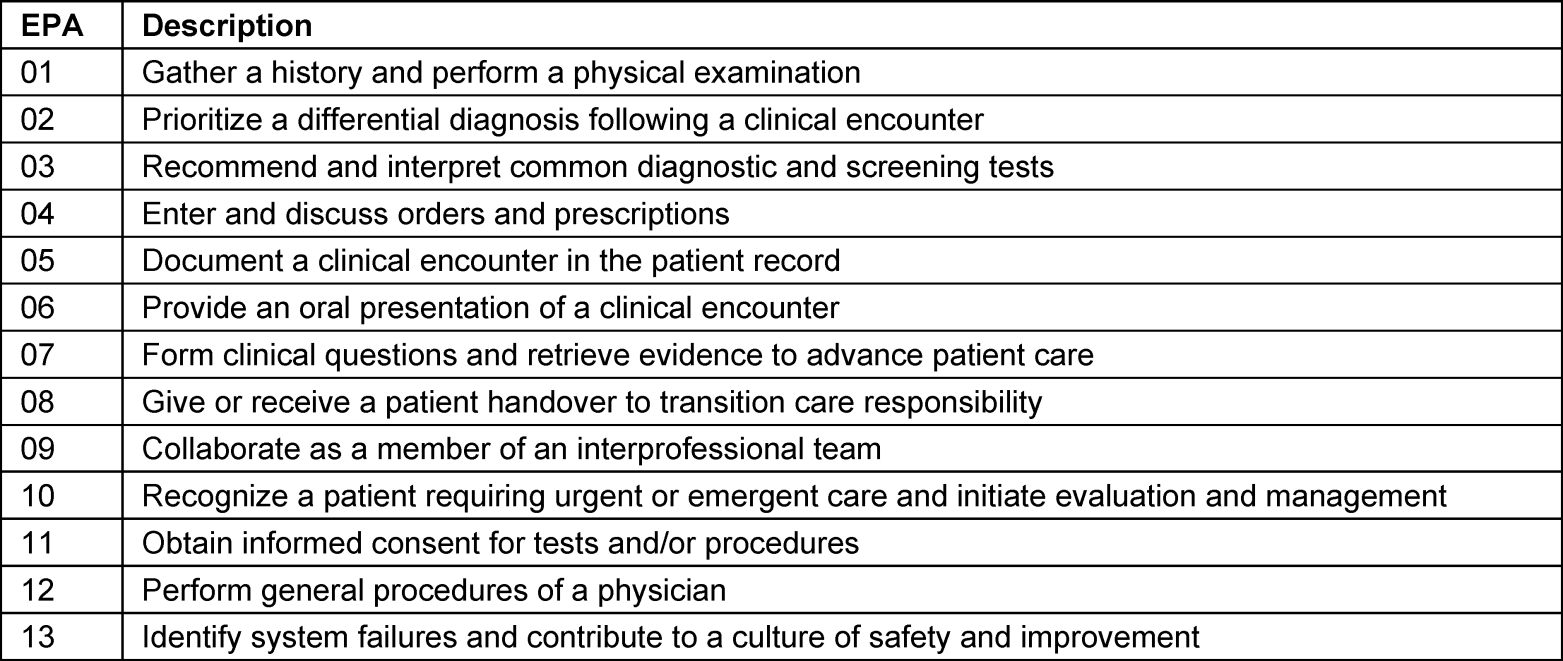
Entrustable Professional Activities [24]

**Table 2:**
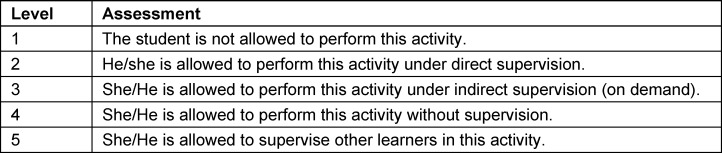
Chen scale [27]

**Table 3:**
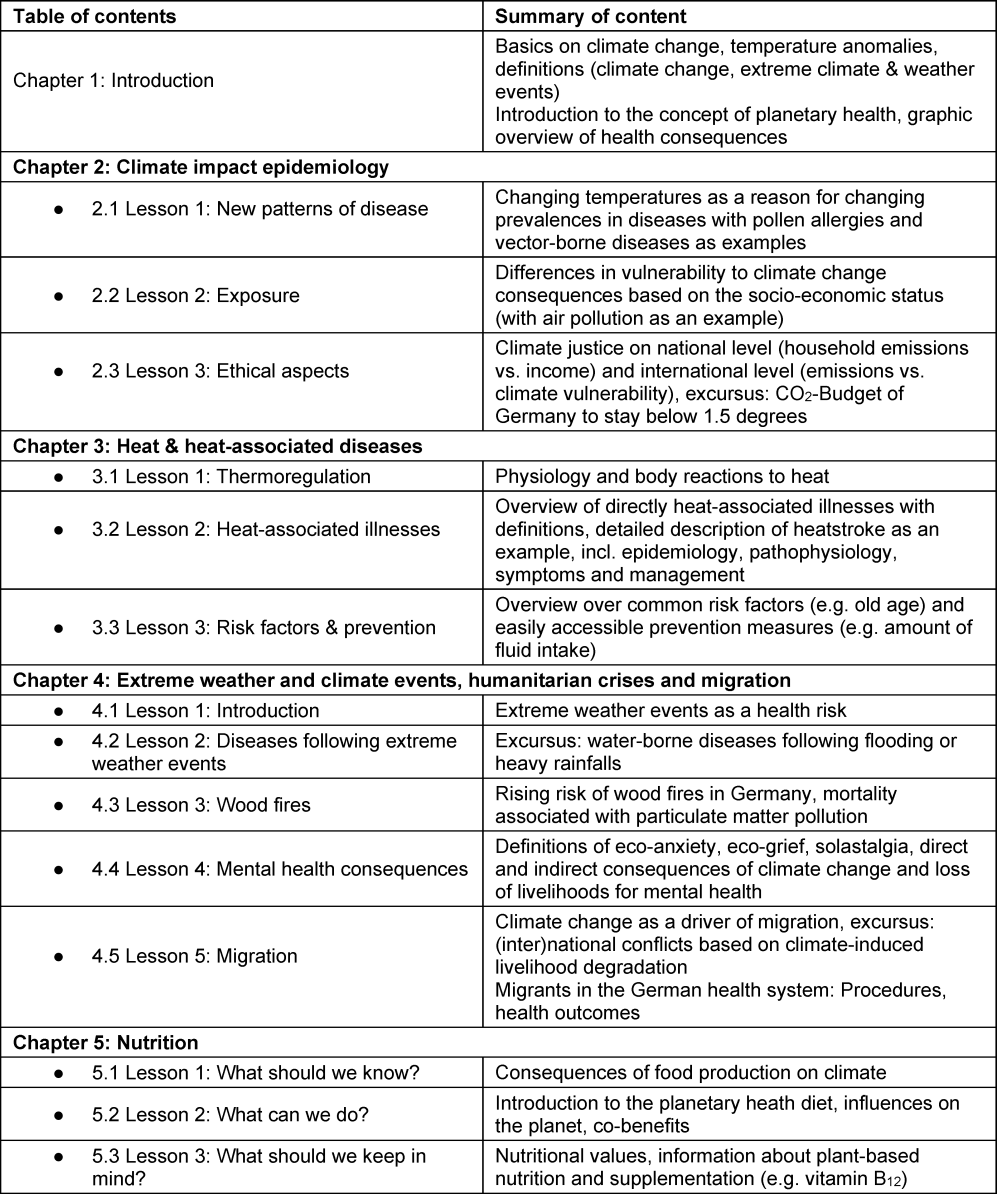
Summary of e-learning content
