# Supplementary material for: Targeting the gap of planetary health education in medical teaching: A student-led initiative develops the course “Klima-LIMETTE” on climate change and health using simulated patients
Source: GMS J Med Educ. 2025 Sep 15;42(4):Doc48. doi: 10.3205/zma001772 (PMC12527391; doi:10.3205/zma001772)
Supplement: Case scenarios [file JME-42-48-s-001.pdf]

Attachment 1: Case scenarios

#	Case	Task	Description	Expected professional activity	EPA
1	Heat Emergency (Heatstroke)	On a sunny afternoon, for which you had planned a visit to the swimming pool, you suddenly receive a worried call from your neighbor: "I'm just coming in the door here, and my husband is not well at all. I don't know him like that. So I thought you were studying medicine...". You immediately make your way to the attic apartment and enter the living room...	The student is called – being a neighbor with a medical background – to the attic apartment of an elderly couple. Upon entering the apartment, he/she notices that it is very hot. In the living room, the husband is found sweaty, covered in blankets and with decreased vigilance (GCS 12-13). The wife expresses measured pathological vital signs (RR 90/60 mmHg, HR 120/min) and is very worried about her husband. She is overwhelmed by the situation but cooperative and eager to help the student. The context of external circumstances, history, vital signs, and clinical examination suggest a heat stroke.	The student should call the ambulance and provide first aid. They can also apply cooling measures, such as undressing the patient or opening the door and have to address the anxious wife, who is supposed to emotionally pressure the student. The student should not try to serve water to the patient, as the patient is at risk of aspiration.	9, 10
2	Mental distress (PTSD)	You are a general practitioner in family doctor's office. A new patient is waiting for you. Conduct the initial interview and decide how to proceed with the patient. Document your intentions (prescription, referral, etc.).	The student is a general practitioner in a family doctor's office. A young patient who has moved from the Ahr valley to Münster after the loss of his/her house comes to him/her for the first time. The patient describes psychosomatic as well as PTSD symptoms (insomnia, flashbacks and recurrent attacks of palpitations and tremors, which, however, have been clarified and have no somatic cause). The patient has lost his/her house and almost all of his/her possessions in the course of the flood in the Ahr valley in 2021.	The student should take a comprehensive history, with a focus on psychosocial condition, and decide how to proceed with the patient (e.g. prescription of psychotropic drugs, psychiatric referral). The exact psychiatric differential diagnosis is not relevant in this scenario but the student should recognize a climate-associated mental distress condition and the need for prompt intervention.	1, 4
3	Vector-borne diseases (Borreliosis)	Take a medical history and perform a physical examination. Formulate a tentative diagnosis based on the information obtained and clarify the patient's questions.	A patient with worsening general condition comes to the general practice. In the further medical history, the patient explains that he/she has recently been on a camping trip in Bavaria. In addition, on close physical examination, an erythema migrans can be found (with pruritus).	The student should take a history and do a physical examination and formulate a tentative suspected diagnosis (on Borreliosis).	1, 2

Attachment 1 to Lilier K, Bärnighausen K, Kuczius T, Jaeger VK, Basoglu A, Karch A, Theiler T, Ajani A, Schwienhorst-Stich EM, Ahrens H. *Targeting the gap of planetary health education in medical teaching: A student-led initiative develops the course "Klima-LIMETTE" on climate change and health using simulated patients.* GMS J Med Educ. 2025;42(4):Doc48. DOI. 10.3205/001772

#	Case	Task	Description	Expected professional activity	EPA
4	Consultation for heat risk prevention	You are a family doctor and your next patient is already waiting for you. You see the name and immediately remember her heart attack two years ago. You have no other information why the patient is presenting today. Nothing else is written about the current reason for the presentation. Evaluate the patient's current complaints and advise her about them.	The student is in the role of a family doctor. The patient introduces him/herself because he/she is worried about the heat, having heard on the radio that it can be harmful to health, especially for people with pre-existing conditions. The social history shows a low socio-economic status, so that there are fewer options for heat prevention (e.g. he/she lives in a non-air-conditioned attic apartment and has no possibility of moving or installing air conditioning).	The student should evaluate the personal risk factors of the patient and give advice about how to minimize them. The advice should be adapted to the patient's situation and be feasible (e.g. visit public air-conditioned rooms, lower shutters during the day and ventilate at night, monitor water supply).	11
5	Nutrition (Co-benefits and risks)	A sports student of physical education comes to your general practice. Perform nutritional counselling with the patient.	A student of physical education consults the general medical practice with the question of transitioning to a plant-based diet. The patient has heard from friends about a vegan diet and would like to try it out, but has also heard from others about various risks of a vegan diet and is therefore concerned. The patient confronts the student with the following "myths":<br>- Vegan diet cannot meet daily protein requirement/nutrient requirement<br>- Vegan diet harms teeth and bones – you have to eat dairy products to meet calcium needs<br>- It is harder to lose weight because you eat a lot of carbohydrates<br>- You have to take a lot of supplements<br>- Vegan diet leads to deficiency symptoms such as fatigue/lack of strength/loss of performance in sports.	The student should provide nutritional advice, explain a plant-based balanced diet and point out health benefits and co-benefits. Since the patient is about to take an exam, it should also be discussed what to watch out for when changing diet and possible substitution options should be mentioned. The "myths" should be reconsidered. The medical history could address any iron deficiency anemias (e.g., due to menstruation). A check of iron/vitamin B12 in the course should also be recommended.	7
6	Allergies to pollen	After a long day of work in your family doctor's office, you look forward to a little walk after work, because although it's only February, it already feels like spring. But first you have to deal with your patients' inquiries on the answering machine. If necessary, make a note of what further steps you would like to take.	In February, the student in the role of a family doctor receives a voice memo on the answering machine. In the memo, a patient aged about 20-40 years describes a runny nose, itchy eyes and a scratchy throat. Due to the symptoms, he/she sleeps less well and becomes drowsy during the day. The patient has no previous illnesses and does not take any medication regularly. The patient asks what he/she can do; above all, he/she would like to be able to sleep better again.	The student should formulate a suspected diagnosis and document further steps, such as specific tests for allergies or medication.	2, 3

#	Case	Task	Description	Expected professional activity	EPA
7	Ethics case	You are a family doctor in an urban practice. You are on your lunch break and have to do your documentation, when you receive an e-mail from the registration office saying “nothing medical”. The work is already piling up next to you. You’re on break and can do whatever you want. If you want to read the e-mail, you will find it printed out in the room. If you want to answer, write your text in the documentary system.	The student receives an email from a patient asking for support in his political engagement. The patient wants to write to the mayor because the particulate matter levels measured in his street are higher than permitted and he suffers from a chronic lung disease. He asks the student for support for his petition and invites him to an event organized by his citizens’ initiative, which is campaigning for traffic calming and urban greening. He asks the student whether he/she could imagine giving a short speech there on the topic of particulate matter and health.	For this case, there is no expected professional activity. Students can ignore the email or reply in as much detail as they wish and determine the level of their commitment themselves.<br>This case intends to be a starting point for discussing role responsibility of the medical professionals in a sociopolitical sense in the seminar.	13
