# Supplementary material for: Targeting the gap of planetary health education in medical teaching: A student-led initiative develops the course “Klima-LIMETTE” on climate change and health using simulated patients
Source: GMS J Med Educ. 2025 Sep 15;42(4):Doc48. doi: 10.3205/zma001772 (PMC12527391; doi:10.3205/zma001772)
Supplement: Analysis of the evaluation of the 1st pilot, winter semester 2022, n=8 [file JME-42-48-s-002.pdf]

Attachment 2: Analysis of the evaluation of the 1st pilot, winter semester 2022, n=8

Climate change and health

	(5) = Strongly agree	(4) = agree	(3) = neutral	(2) = disagree	(1) = Strongly disagree	n=
**1.** The course has increased my interest in the topic of climate change and health.	75%	25%	0%	0%	0%	8
**2.** The course has increased my interest in the topic of climate change and health.	62,5%	25%	12,5%	0%	0%	8
**3.** The course made me curious to engage further with the topic.	12,5%	87,5%	0%	0%	0%	8
**4.** My opinion of a specific topic within the course has changed.	12,5%	25%	25%	37,5%	0%	8
**5**. Climate change impacts our clinical work and patients.	62,5%	37,5%	0%	0%	0%	8

Attachment 2 to Lilier K, Bärnighausen K, Kuczius T, Jaeger VK, Basoglu A, Karch A, Theiler T, Ajani A, Schwienhorst-Stich EM, Ahrens H. *Targeting the gap of planetary health education in medical teaching: A student-led initiative develops the course “Klima-LIMETTE” on climate change and health using simulated patients.* GMS J Med Educ. 2025;42(4):Doc48. DOI. 10.3205/001772

Preperation for the course

	(5) = Strongly agree	(4) = agree	(3) = neutral	(2) = disagree	(1) = Strongly disagree	n=
**1.** The material in the e-learning course was comprehensible.	37,5%	62,5%	0%	0%	0%	8
**2.** The amount of the material for preparation was appropriate.	0%	75%	12,5%	12,5%	0%	8
**3.** The material was presented in a way that highlighted links between the topics.	0%	75%	12,5%	12,5%	0%	8
**4.** The presented media (Graphs, videos) help me to understand the topic.	12,5%	75%	12,5%	0%	0%	8
**5**. Die e-learning course has prepared me sufficiently for the simulation.	0%	62,5%	37,5%	0%	0%	8

6. Amount of time used for the e-learning course: 30 min (n=1), 60 min (n=2), 70 min (n=2), 90 min (n=2), 120 min (n=1)

Simulation

	(5) = Strongly agree	(4) = agree	(3) = neutral	(2) = disagree	(1) = Strongly disagree	n=
**1.** The cases were realistic and of clinical relevance.	75%	25%	12,5%	0%	0%	8
**2.** The solving of some cases was overwhelming for me	0%	33,3%	50%	16,6%	0%	6

Which one(s)? Nutrition counseling (n=2), heat emergency, heat education

**3.** The simulations taught me something new.	50%	50%	0%	0%	0%	8
**4.** The simulations make me more confident in dealing with climate associated health conditions.	50%	37,5%	12,5%	0%	0%	8

Seminar

	(5) = Strongly agree	(4) = agree	(3) = neutral	(2) = disagree	(1) = Strongly disagree	n=
**1.** The seminar helped me to deepen my understanding of the course topics.	37,5%	62,5%	0%	0%	0%	8
**2.** The seminar encouraged my to think critically about the taught topics.	37,5%	62,5%	0%	0%	0%	8

Attachment 2 to Lilier K, Bärnighausen K, Kuczius T, Jaeger VK, Basoglu A, Karch A, Theiler T, Ajani A, Schwienhorst-Stich EM, Ahrens H. *Targeting the gap of planetary health education in medical teaching: A student-led initiative develops the course “Klima-LIMETTE” on climate change and health using simulated patients.* GMS J Med Educ. 2025;42(4):Doc48. DOI. 10.3205/001772

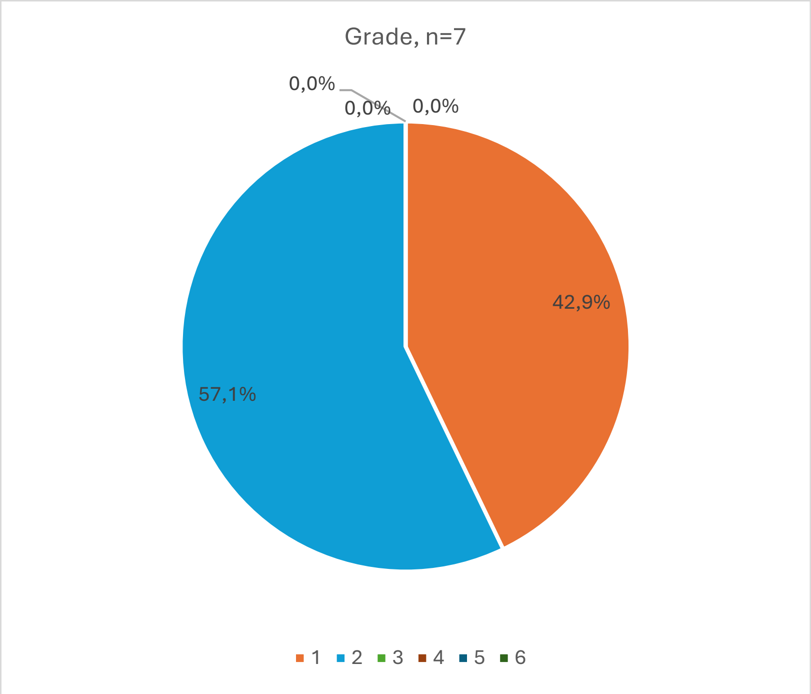

Personal feedback

Do you have suggestions for improvement? What did you like/dislike?
Participant 1: Interactive exercises of the e-learning course weren't doable on a tablet. Background was comprehensibly explained, for a few cases I was missing diagnostic and therapy in the e-learning course.<br><br>Participant 2: Graphics gave great overviews in the e-learning course.<br><br>Participant 4: Thanks, it was fun, the cases were close to reality.<br><br>Participant 5: Didn't like the opinion poll in the e-learning, no yes/no- questions<br><br>Participant 6: More case-specific, content-based discussion in the seminar<br><br>Participant 7: Delete headlines of cases, they give away too much beforehand.<br><br>Participant 8: Preparation materials were partly incomplete and very extensive. The discussion of the cases in the seminar was very helpful and has highlighted important aspects of the cases.

Which grade do you give the course? (1=very good - 6=deficient)
