# Supplementary material for: Targeting the gap of planetary health education in medical teaching: A student-led initiative develops the course “Klima-LIMETTE” on climate change and health using simulated patients
Source: GMS J Med Educ. 2025 Sep 15;42(4):Doc48. doi: 10.3205/zma001772 (PMC12527391; doi:10.3205/zma001772)
Supplement: Analysis of the evaluation of the 2nd pilot, 1st trail as elective course, summer semester 2023, n=24 [file JME-42-48-s-003.pdf]

# Attachment 3: Analysis of the evaluation of the 2nd pilot, 1st trail as elective course, summer semester 2023, n=24

Pre- & post-evaluation questions:

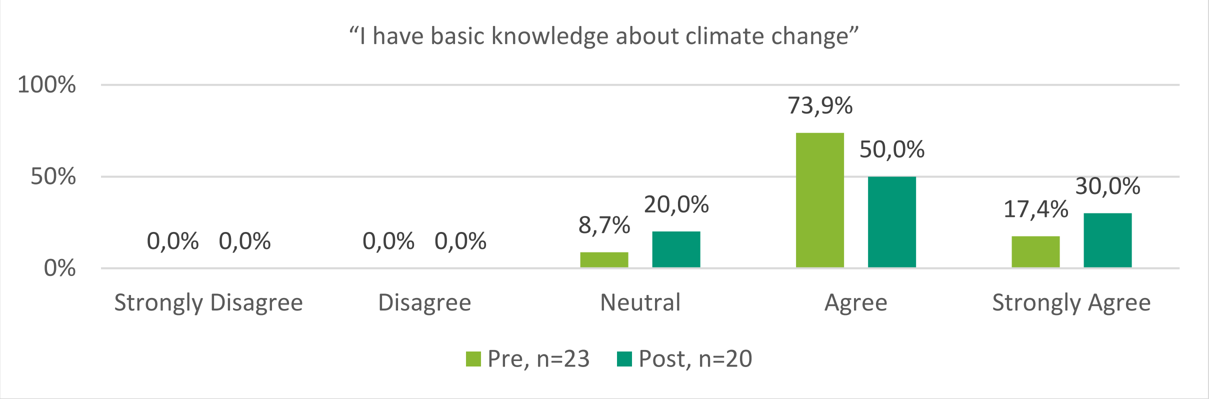


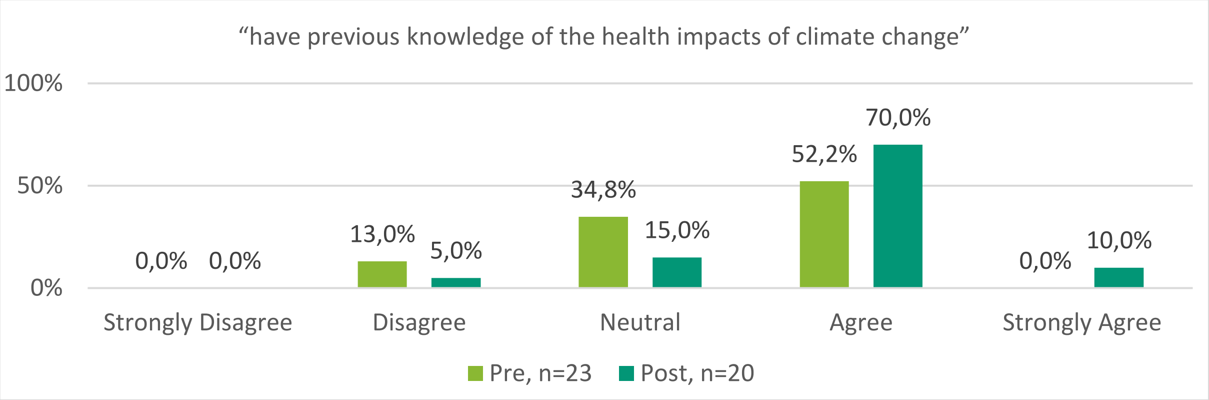


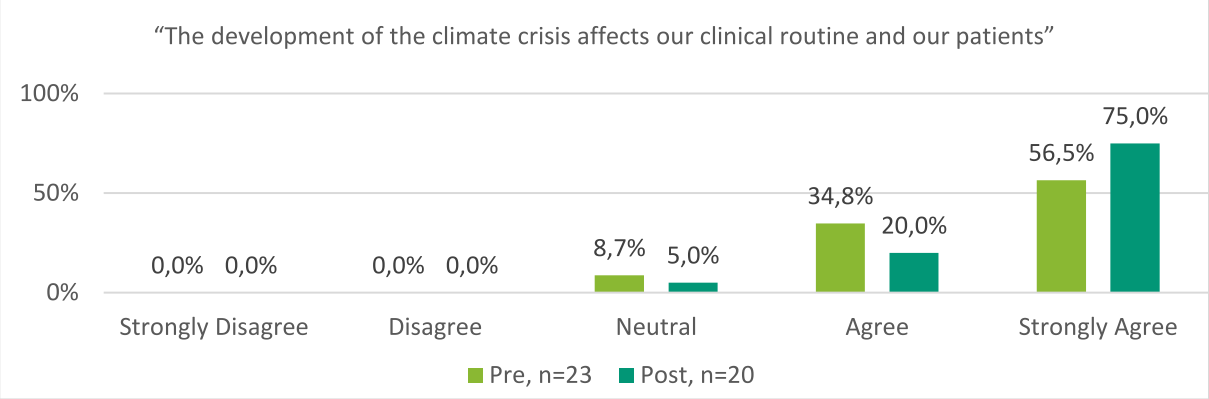


Attachment 3 to Lilier K, Bärnighausen K, Kuczius T, Jaeger VK, Basoglu A, Karch A, Theiler T, Ajani A, Schwienhorst-Stich EM, Ahrens H. *Targeting the gap of planetary health education in medical teaching: A student-led initiative develops the course "Klima-LIMETTE" on climate change and health using simulated patients.* GMS J Med Educ. 2025;42(4):Doc48. DOI. 10.3205/001772

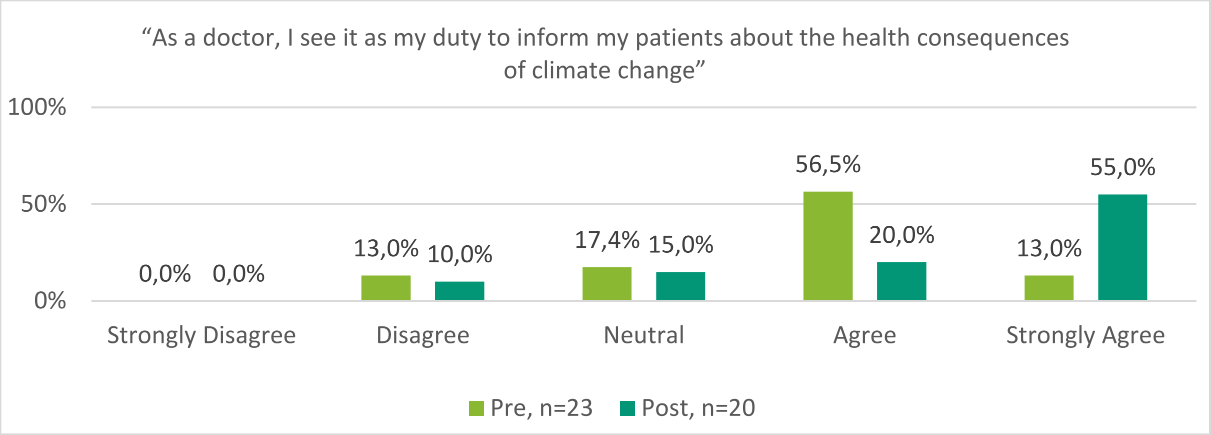
"As a doctor, I see it as my duty to inform my patients about the health consequences of climate change"
100%
50%
0%
0,0%
0,0%
13,0%
10,0%
17,4%
15,0%
56,5%
20,0%
13,0%
55,0%
Strongly Disagree
Disagree
Neutral
Agree
Strongly Agree
Pre, n=23
Post, n=20

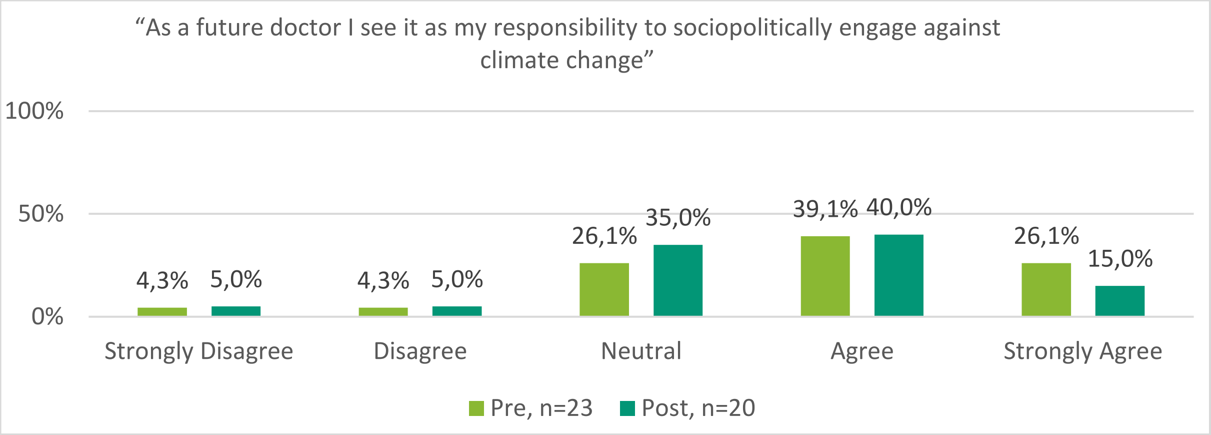
"As a future doctor I see it as my responsibility to sociopolitically engage against climate change"
100%
50%
0%
4,3%
5,0%
4,3%
5,0%
26,1%
35,0%
39,1%
40,0%
26,1%
15,0%
Strongly Disagree
Disagree
Neutral
Agree
Strongly Agree
Pre, n=23
Post, n=20

Post-evaluation:

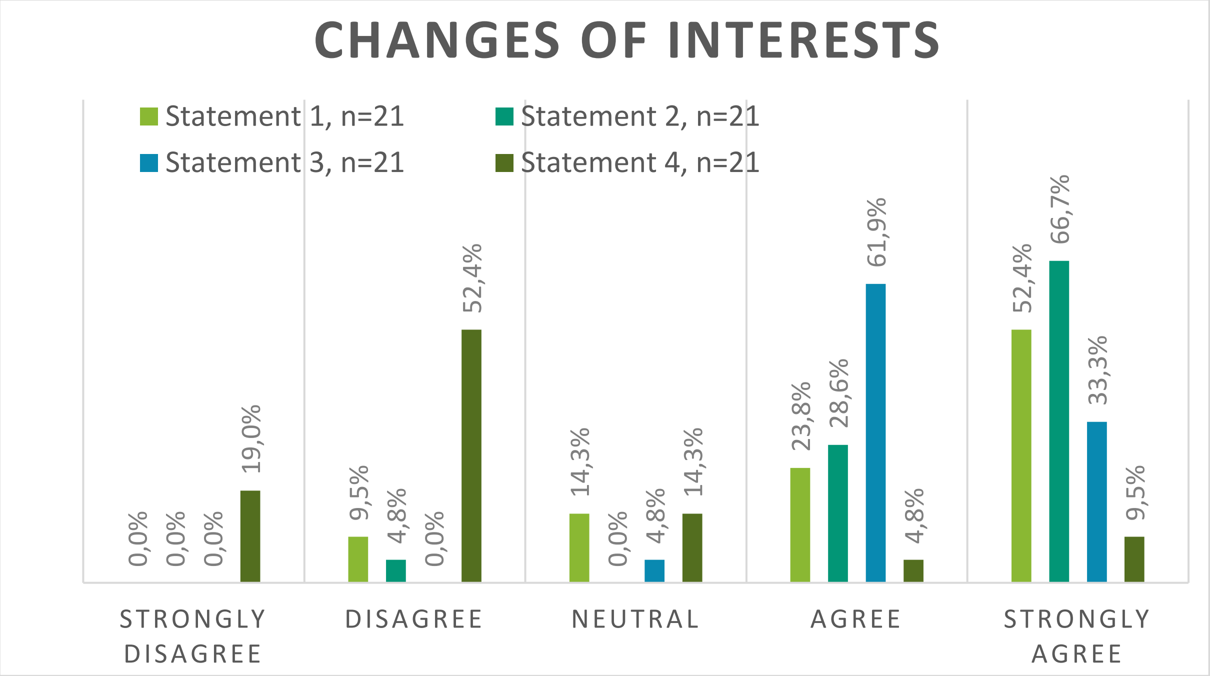


Statement 1: I was already interested in the topic of climate change and health before I took part in the course.

Statement 2: The course has awakened/reinforced my general interest in the topic.

Statement 3: The course made me curious about a further examination of the teaching content.

Statement 4: My opinion on a certain topic of the course has changed.

Specifications on Statement 4: My opinion on a certain topic of the course has changed, n=4.

“On the acute relevance of the topic in clinical work! Before that, I always thought primarily about the global effects and the future, but I didn't think about the heat waves and the consequences of the flood in the Ahr Valley etc. and therefore related it less to the clinical work in Germany.”

“That concern for the future of the planet can manifest itself in the form of a clinically recognized mental illness.”

“Nutrition”

“Extent of the influence of climate change on social inequality”

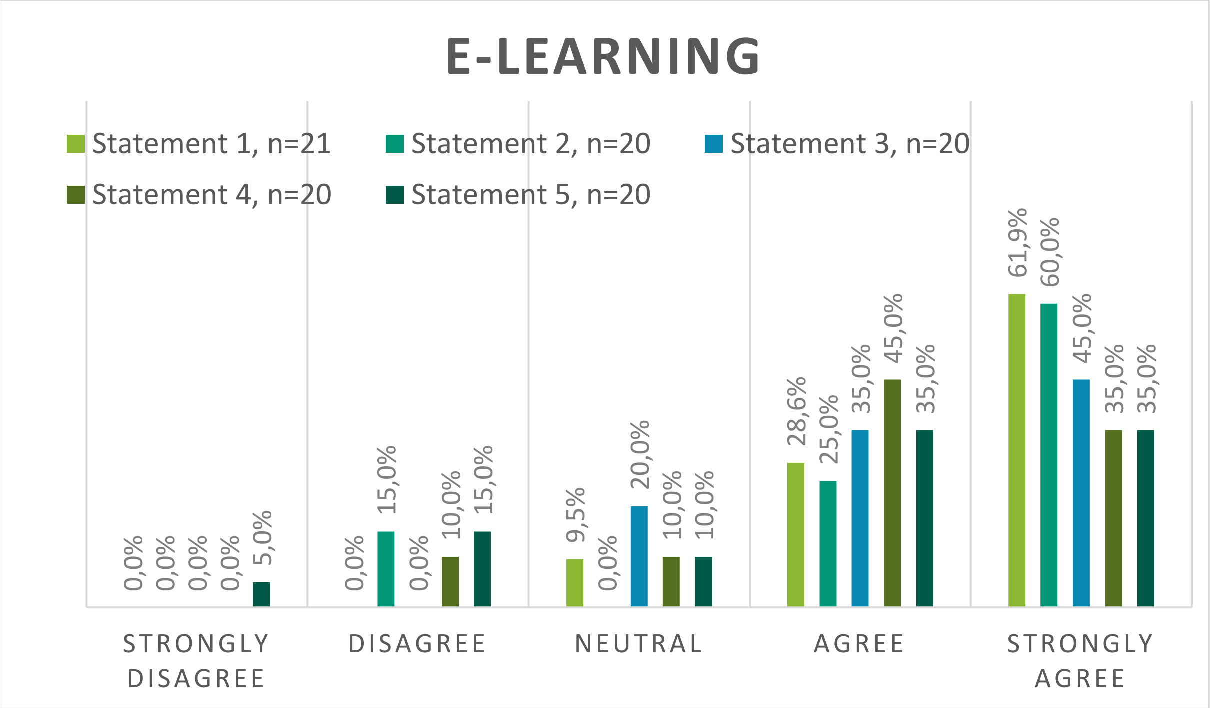


Statement 1: The material in the e-learning was comprehensible.

Statement 2: The amount of material provided for preparation was adequate.

Statement 3: The content was presented in such a way that cross-connections between the topics covered became clear to me.

Statement 4: The media used (graphics, videos) helped me to understand the content conveyed.

Statement 5: The e-learning prepared me sufficiently for working on the cases simulations.

Attachment 3 to Lilier K, Bärnighausen K, Kuczius T, Jaeger VK, Basoglu A, Karch A, Theiler T, Ajani A, Schwienhorst-Stich EM, Ahrens H. *Targeting the gap of planetary health education in medical teaching: A student-led initiative develops the course "Klima-LIMETTE" on climate change and health using simulated patients.* GMS J Med Educ. 2025;42(4):Doc48. DOI. 10.3205/001772

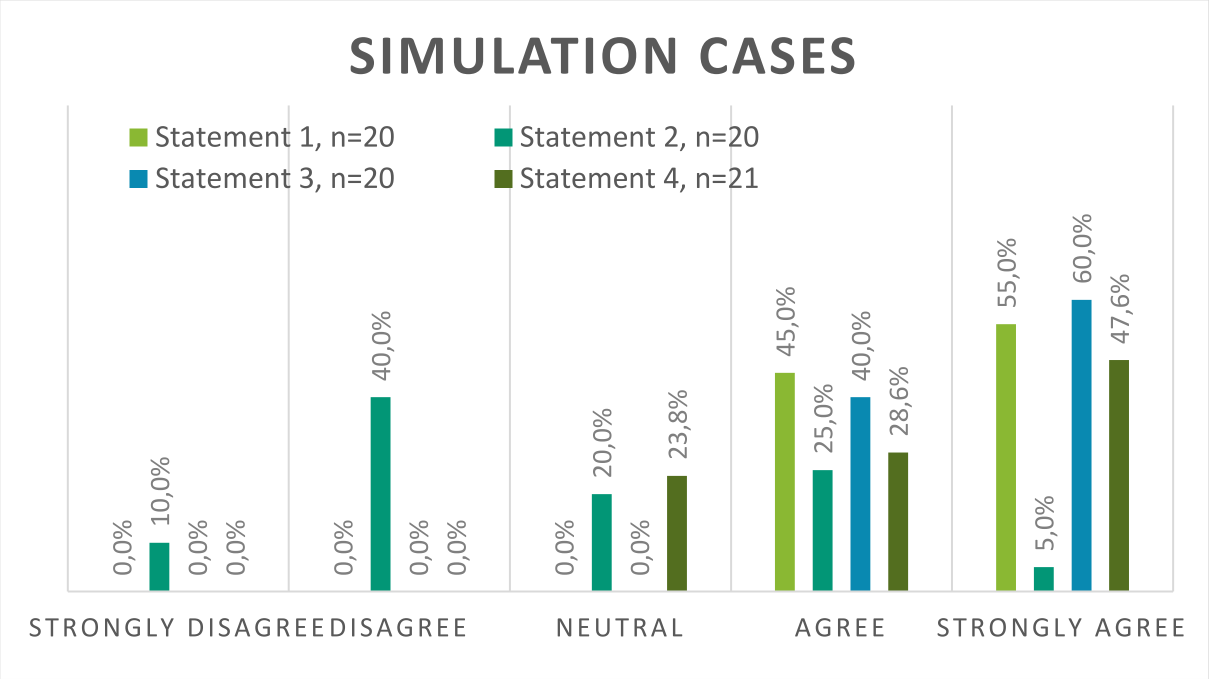


Statement 1: The simulation cases were selected to be realistic and clinically relevant.

Statement 2: Solving some cases overwhelmed me.

Statement 3: I learned something new by participating in the course.

Statement 4: I feel better trained in dealing with patients with climate-related health problems thanks to this course

Specifications on Statement 2: Solving some cases overwhelmed me, n=10.

“Nutritional counseling was faltering” [case 5]

“Heat stroke” [case 1]

“Only the emergency was relatively difficult because it was not completely recognizable in the setting. Perhaps more details could have been written on the door, i.e. temperature in the room, temperature of the patient, etc. And I have inhibitions about actually using water etc. in the drama setting - perhaps you could have put an empty bucket etc. there and said as an assignment: PLAY what you WOULD do?” [case 1]

“mainly advice, but everything else too”

“Counseling sessions on vegan nutrition and heat patient” [case 5 & 4]

“I still lacked confidence in dealing with emergencies” [case 1]

“The heat stroke, but for formal reasons. I was initially confused for quite a while whether the patient had a heatstroke at all, because the LIMETTE conditions did not indicate an increased temperature load (perhaps the partner made it clearer that he was so overheated). It was also unclear to me during the situation how far the actors should be exposed: Would it be OK to strip the actors down to their underwear? (also applies to the patient with Lyme disease) To put wet towels on his body? If 24 people do that one after the other, you'll get cold at some point. With the cold packs, it would also be more practical if it was somehow clear that they were available in the room. But maybe I just wasn't engrossed enough in the acting situation”. [case 1]

“Nutritional advice, Lyme disease” [case 5 & 3]

“Woman with worries from a socially disadvantaged background” [case 4]

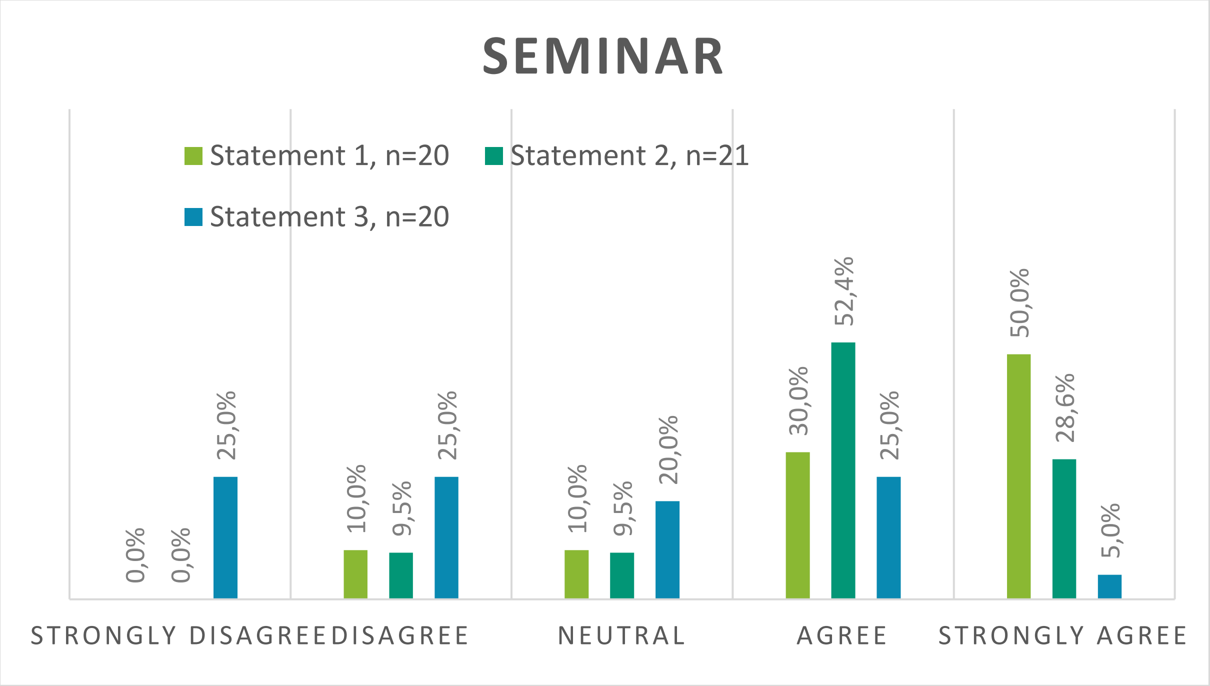


Statement 1: The seminar deepened my understanding of the content.

Statement 2: The seminar encouraged me to take a critical look at the topic.

Statement 3: I would have preferred to use the entire seminar time to talk about the simulation cases.

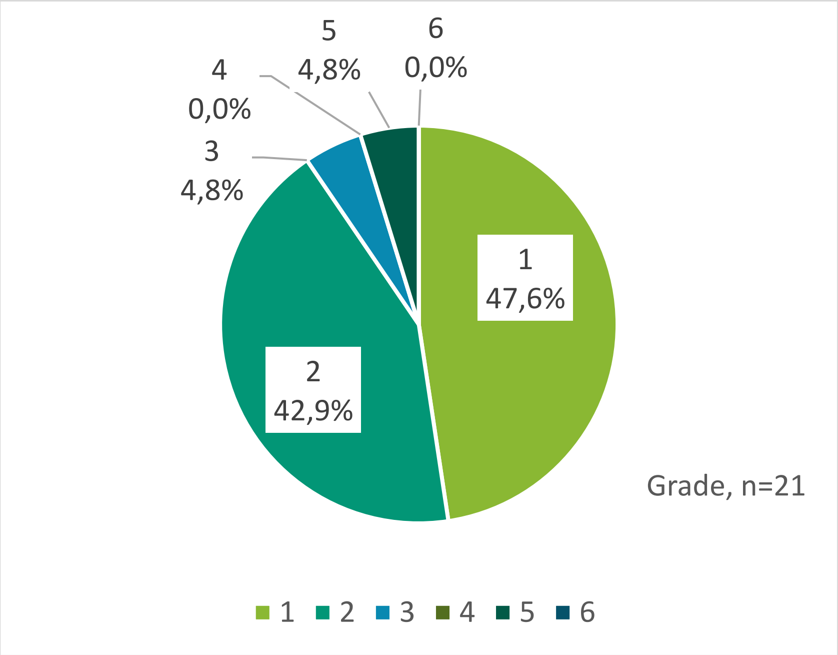

Grading (1=very good- 6=deficient)

Improvement suggestions and general feedback, n=7:

Participant 1: Particularly good: Nutrition counseling case. Would love to see more on sustainable healthy eating.

Participant 2: More simulation cases! :D I found counseling easy, but the emergency was very challenging, and I would have liked to practice more chaotic/confusing situations (e.g. person directly after forest fire/flooding/earthquake?).

Participant 3: I thought the course was really good and have no real suggestions for improvement! In the seminar, it became clear that the actors didn't all act the same, so homogenizing the scripts would probably be helpful. And as I said above, in the emergency (heat stroke) I would have liked more details about the patient at the door and a clear order to *act*, because I wouldn't dare to use real water on the actor in that setting... But it was really great, especially with the pause [Ethic case], which was very thought-provoking. Thank you very much!

Participant 4: As I'm still quite early in my studies, I have little prior knowledge and it was overwhelming for me. I would like to see more procedures in the preparation, e.g. for diagnosis, therapy etc.

Participant 5: I sometimes couldn't understand the EPAs and sometimes got 0 points.

Participant 6: It was by far the best LIMETTE-course for me so far! I found the cases very appropriate and your preparation and guidance super! Thank you very much.

Participant 7: Overall, a great format and a great elective! 2 more ideas: More time between the cases to take notes. I think it should have been 2 minutes, but in practice we all had to move on seamlessly. A few more details about the diseases and their treatment in the e-learning. With infectious diseases in particular, you're ill-prepared if you haven't had the material before or if it was covered too long ago.
